# Increase in Brain Volume After Aneurysmal Subarachnoid Hemorrhage Leads to Unfavorable Outcome: A Retrospective Study Quantified by CT Scan

**DOI:** 10.3389/fneur.2021.654419

**Published:** 2021-10-08

**Authors:** Bin Qin, Yi Xiang, Jianfeng Zheng, Rui Xu, Zongduo Guo, Chongjie Cheng, Li Jiang, Yue Wu, Xiaochuan Sun, Zhijian Huang

**Affiliations:** ^1^Department of Neurosurgery, The First Affiliated Hospital of Chongqing Medical University, Chongqing, China; ^2^Department of Neurosurgery, The Second People's Hospital of Jiulongpo District, Chongqing, China; ^3^Department of Neurosurgery, Chongqing University Central Hospital, Chongqing, China

**Keywords:** clinical prognosis, brain volume, 3D-slicer, primary brain swelling, aneurysm subarachnoid hemorrhage

## Abstract

**Background and Purpose:** Primary brain swelling occurs in aneurysmal subarachnoid hemorrhage (aSAH) patients. The absence of a dynamic quantitative method restricts further study of primary brain swelling. This study compared differences in the change rate of brain volume (CRBV) between patients with and without primary brain swelling in the early stage of aSAH. Moreover, the relationship between CRBV and clinical outcomes was evaluated.

**Methods:** Patients hospitalized within 24 h after aSAH were included in this retrospective study. Utilizing a qualitative standard established before the study to recognize primary brain swelling through brain CT after aSAH, clinical outcomes after 3 months of SAH were evaluated with a modified Rankin scale (mRS). The brain volume (BV) of each patient was calculated with a semiautomatic threshold algorithm of 3D-slicer, and the change in brain volume (CIBV) was obtained by subtracting the two extreme values (CIBV = BV_max_ – BV_min_). The CRBV was obtained by CIBV/BV_min_ × 100%. The CRBV values that predicted unfavorable prognoses were estimated.

**Results:** In total, 130 subjects were enrolled in the study. The mean CRBV in the non-swelling group and swelling group were 4.37% (±4.77) and 11.87% (±6.84), respectively (*p* < 0.05). CRBV was positively correlated with the length of hospital stay, blood in the ambient cistern, blood in the lateral ventricle, and lateral ventricular volume (Spearman ρ = 0.334; *p* < 0.001; Pearson ρ = 0.269, *p* = 0.002; Pearson ρ = 0.278, *p* = 0.001; Pearson ρ = 0.233, *p* = 0.008, respectively). Analysis of variance showed significant differences in CIBV, CRBV, blood in the ambient cistern, blood in the lateral ventricle, and lateral ventricular volume among varying modified Fisher scale (mFisher), with higher admission mFisher scale, indicating larger values of these variables. After adjusting for risk factors, the model showed that for every 1% increase in the CRBV, the probability of poor clinical prognosis increased by a factor of 1.236 (95% CI = 1.056–1.446). In the stratified analysis, the odds of worse clinical outcomes increased with increases in the CRBV. Receiver operating characteristic curve analysis showed that HH grade, mFisher scale, and score of CRBV (SCRBV) had diagnostic performance for predicting unfavorable clinical outcomes.

**Conclusion:** Primary brain swelling increases brain volume after aSAH. The CRBV quantified by 3D-Slicer can be used as a volumetric representation of the degree of brain swelling. A larger CRBV in the early stage of aSAH is associated with poor prognosis. The CRBV can be used as a neuroimaging biomarker of early brain injury after bleeding and may be an effective predictor of patients' clinical prognoses.

## Introduction

Aneurysmal subarachnoid hemorrhage (aSAH) is a devastating acute cerebrovascular disease that causes high mortality and morbidity (1, 2). Although the survival rates of aSAH have increased in the last several decades because of advanced high-tech diagnostic and treatment technologies, aSAH is still the major factor of stroke-related death (3). Older age, worse clinical classification at presentation, large aneurysm size, aneurysm rebleeding, and cerebral infarction from vasospasm are well-established risk factors for mortality (4, 5). Brain swelling can be found very early after bleeding in patients with aSAH and can be divided into primary brain swelling after ictus and secondary brain swelling due to complications (e.g., infarction, hemorrhage, hydrocephalus). Whether the etiology is primary or secondary, brain swelling will aggravate the prognosis after aSAH, and its drug treatment may be related to serious side effects (6). The efficacy and indication of decompressive hemicraniectomy (DHC), which is regarded as a last resort remedy for brain swelling after failure of conservative treatment, is still disputed (7). A few data refer to the impact of the etiology of the volume effect, which results in DHC that might significantly affect prognosis (8). Therefore, the accurate identification and effective treatment of brain swelling in the early stage of aSAH may help patients avoid DHC. Currently, the mechanism of primary brain swelling is largely unknown. The main imaging indications of brain swelling are narrowing and disappearance of the cerebral sulcus and basal cisterns (6). Brain swelling has a substantial impact on neurological function and quality of life of the sufferers (9). However, the current diagnostic criterion of brain swelling is qualitative and relies mainly upon the clinical diagnosis of the neurosurgeon, which is not conducive to assessing the severity of brain swelling and its relationship with clinical outcomes. The degree of brain swelling is a subjective description. As a result, the absence of reliable quantification measurements hinders further judgment and exploration of the risk factors for primary brain swelling. The success of quantitative imaging depends on robust analysis software tools and methods. 3D-Slicer is a volumetric analysis software widely used in neurosurgery, and various types of medical neuroimaging data can be processed, analyzed, and visualized using a featured module (10, 11). In this research, we developed a new quantitative approach based on threshold technology to measure brain volume (BV) and the change rate of brain volume (CRBV) through 3D-Slicer. We hypothesize that the CRBV obtained in the early period of aSAH is a volumetric presentation of the varying degrees of primary brain swelling. In addition, CRBV may be an alternative neuroimaging biomarker of early brain injury (EBI), which could predict the clinical outcomes of patients with aSAH.

## Subjects and Methods

### Participants

**Figure 1:**
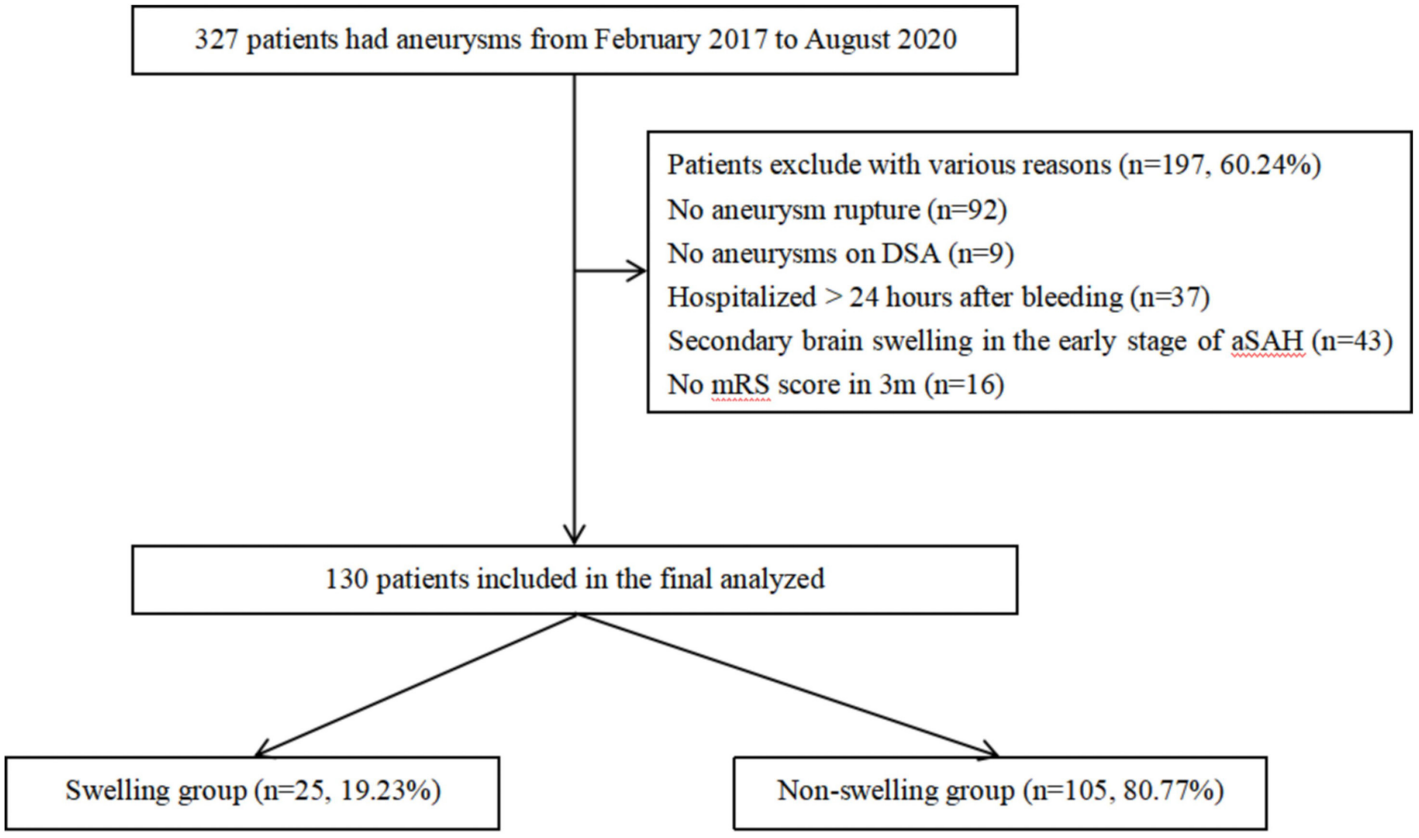
A total of 327 patients with intracerebral aneurysms between February 2017 and August 2020 were collected. After selection, 130 aSAH patients were included. aSAH; aneurysms subarachnoid hemorrhage.

Patients diagnosed with aSAH and hospitalized in the Neurosurgery Intensive Care Unit (NSICU) of the First Affiliated Hospital of Chongqing Medical University from February 2017 to August 2020 were recorded. This study was approved by the Institutional Review Board of The First Affiliated Hospital of Chongqing Medical University, in accordance with the Declaration of Helsinki. Due to the retrospective nature of the study, the need for informed consent was waived. In our analysis, the subject had to meet the following criteria: (1) aSAH diagnosed by brain CT, CT angiography (CTA), or digital subtraction angiography (DSA); (2) hospitalized within 24 h after bleeding; (3) brain CT performed more than two times in the early stage of SAH (within 72 h after bleeding); and (4) clinical outcome evaluation performed at 3 months after onset. Patients with SAH caused by trauma, arteriovenous malformation rupture, isolated cortical SAH, vasculitis, or patient with a past medical history of craniocerebral injury and related chronic disease; patients with secondary brain swelling caused by cerebral ischemia, rebleeding after aSAH, intracranial infection, and other complications after aSAH; and patients whose overall condition was poor and the estimated survival time was <1 year were eliminated. Finally, 130 subjects were recruited for our research study using our criteria (Figure 1).

### Clinical Variables

We recorded baseline demographic data (age, sex, and ethnicity), previous medical history (hypertension, hyperlipidemia, diabetes, and neurological disease), social history (smoke, alcohol, and drug use), and clinical features on admission [Hunt–Hess grade (12), World Federation of Neurosurgical Societies, modified Fisher scale, loss of consciousness (13)]. The neurological and general medical estimations of all subjects were executed by two neurologists on admission. The therapeutic schemes of all patients were performed according to available guidelines (14). The clinical outcomes of the patients at 3 months after hemorrhage were evaluated by the modified Rankin scale (mRS), and severe disability or death was identified as a mRS ≥ 3 (15).

### Radiographic Variables

Brain swelling was diagnosed when both of the following were present on brain CT: (1) narrowing or disappearance of the cerebral sulcus and basal cisterns; (2) contraction of the ventricle systems, including the lateral ventricle, third ventricle, or fourth ventricle. After blinding to the clinical conditions, brain CT scans of each patient were assessed and judged for the presence or absence of brain swelling by two neurologists. According to whether primary brain swelling occurred in the early stage of aSAH, all subjects were separated into two groups: (a) the swelling group (*n* = 25) and (b) the non-swelling group (*n* = 105). Delayed cerebral ischemia (DCI) was defined as the deterioration of clinical symptoms caused by vasospasm (such as hemiplegia, aphasia, apraxia, hemiblindness, loss of consciousness, etc.), or a new cerebral infarction related to vasospasm that was not visible at admission or on post-operative brain CT, or both at 72 h after ictus of SAH (16). We only identified DCI patients after strictly excluding other possible causes.

### Module in 3D-Slicer

**Figure 2:**
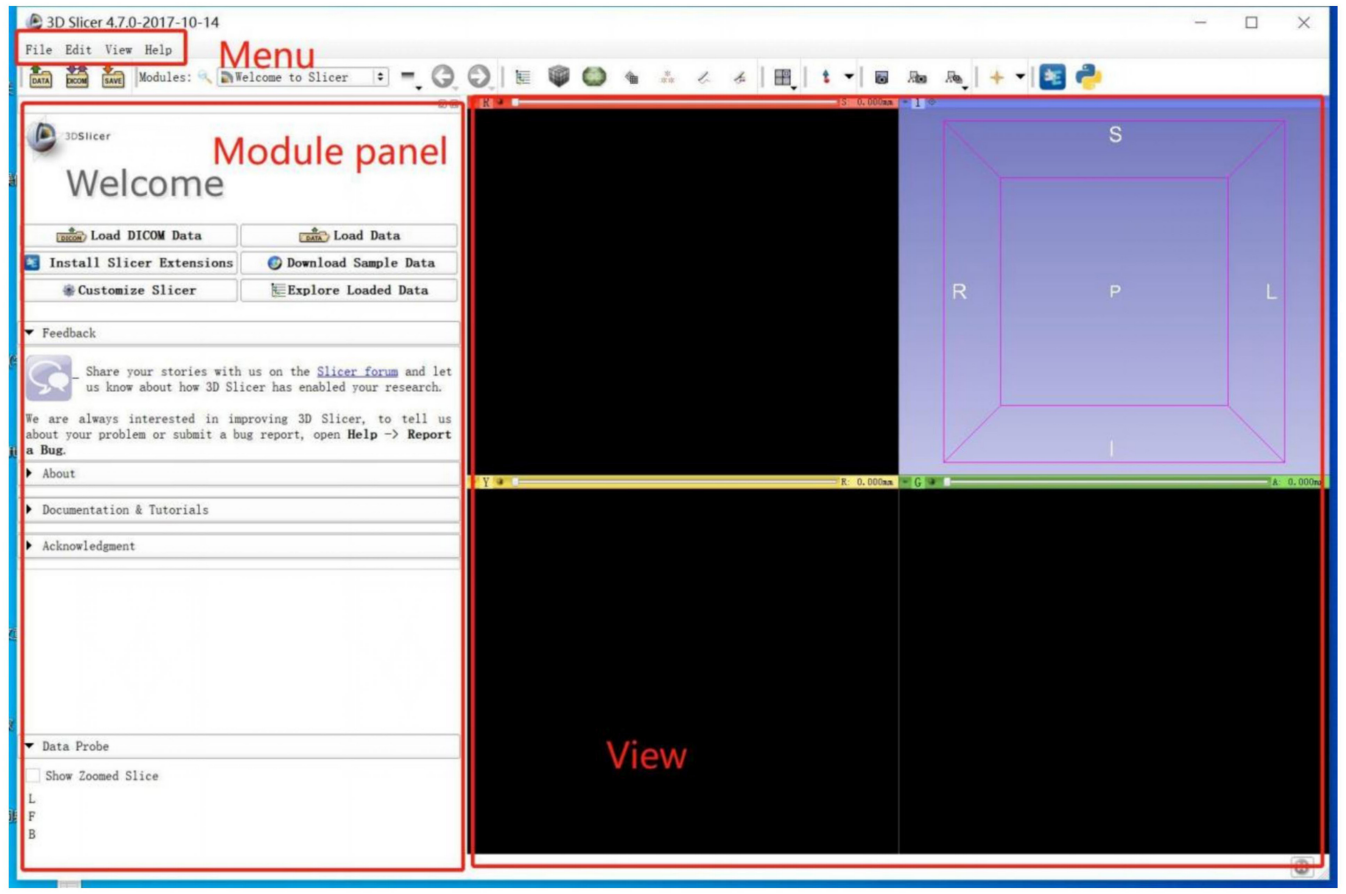
The interface of 3D-Slicer software (version 4.10.2).

3D-Slicer is an open platform that provides several modules. In the scene, different modules can be used to carry out specific algorithms for building or manipulating neuroimaging data (Figure 2). Segment Editor (SE) is a common volumetric segmentation module that is used to draw constructures of the interesting regions. Watershed is an edge-based segmentation tool in SE and an effective way to segment targeted areas. There are some similarities between the algorithms in Watershed and Grow-Cut (17). In the process of segmentation, similarities in adjacent pixels are used as an important reference such that the pixels with similar spatial positions and similar gray values can be connected to form a closed contour (18). The closeness is an important feature of the Watershed algorithm (19); other image segmentation methods do not consider the concepts of the similarity and closeness of the pixels in terms of their spatial relationship, which are independent of each other and cannot be combined. Compared with other segmentation methods, the Watershed algorithm is more ideological and more in line with the evaluation of images by the human eye.

### Image Data Processing

**Figure 3:**
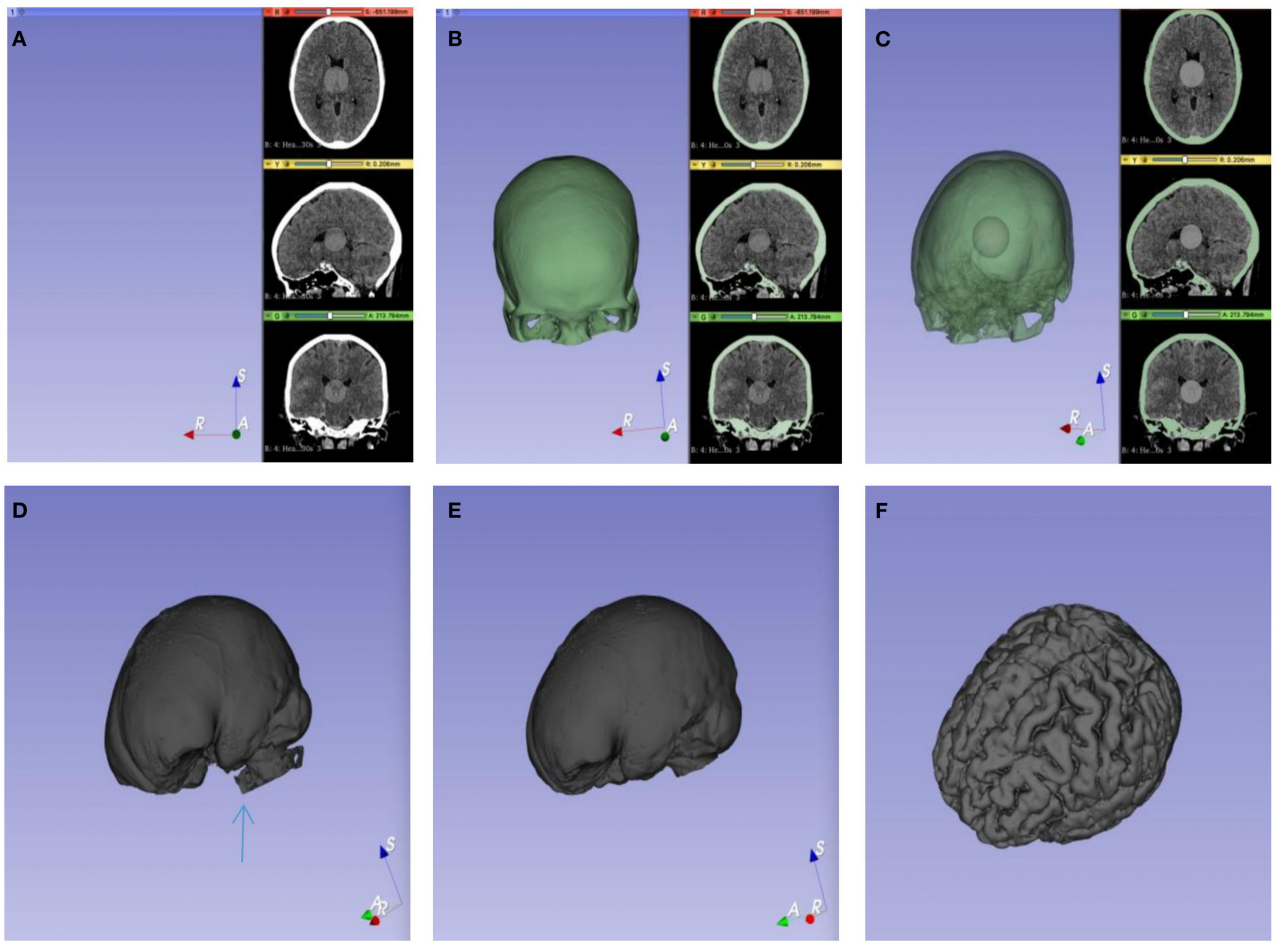
Calculation process of brain volume (BV). **(A)** A gray sphere was drawn in the center of the cranial cavity as the initial value. **(B)** The inner surface of the skull was defined as the outer boundary of the target segmentation image. **(C)** The initialization of segmentation started from the initial value and then iteratively filled. **(D)** The pore structure of the skull base is shown, and a little pixel leakage may be occurred (blue arrow). **(E)** The volume of the cranial cavity was obtained after the leakage was removed with tools. **(F)** Pixel threshold technology was used to remove non-brain tissue.

**Figure 4:**
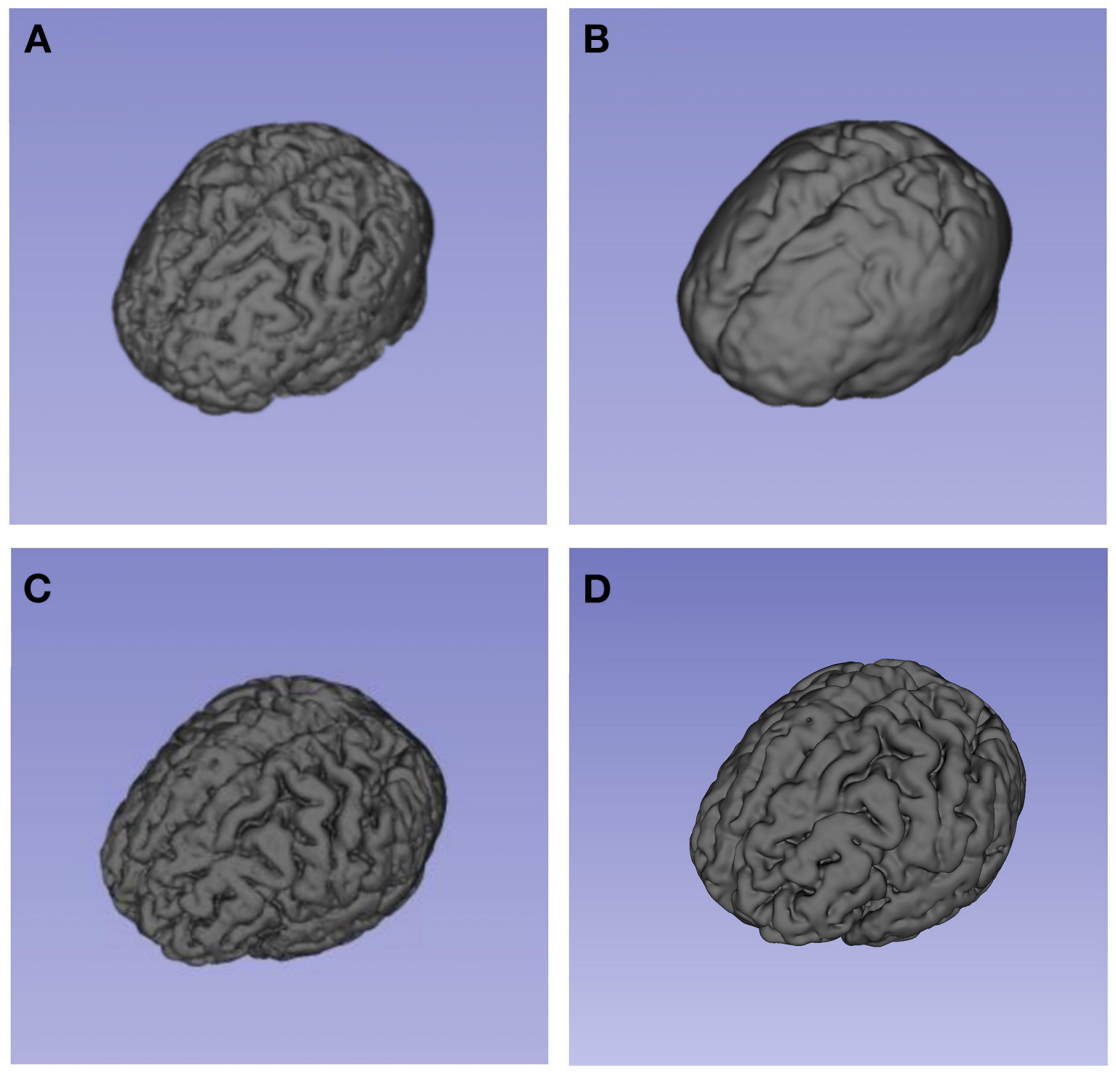
These models present the segmentation result (brain volume) of 3D-Slicer. **(A)** and **(B)** showed the brain volume of a patient in brain swelling group at different time points. **(A)** showed his brain volume within 24 h after bleeding. As shown in **(B)**, the patient developed typically brain swelling with the disease progressed. This patient's CRBV was 11. 35%. By comparison, **(C)** and **(D)** showed the brain volume of a patient in non-brain swelling group. The patient's CRBV was 3.26%.

Brain CT scans were quantified using 3D-Slicer version 4.10.2 (11). Quantitative measurements were carried out based on the threshold algorithm (20). We coded all the data before the calculations of the analysts to eliminate bias. Brain CT scans were collected in the form of Digital Imaging and Communications in Medicine (DICOM) or, infrequently, as hard copies. Then, the data were transferred to the imaging laboratory. The measurements were performed using the following steps for each CT scan: an original label map was created as the input for the watershed effect. A sphere, namely, the original label map, was painted in the center of the intracranial cavity with one color by the paint effect. The semiautomated pixel thresholding technique was used to label the skull. Meanwhile, the skull was painted with another color. Then, the procedure automatically defaulted the exterior edge of the targeted regions as the internal surface of the skull, which can prevent leakage of the label map. In the center of the intracranial cavity, a sphere was created to execute the initialization of segmentation, which fills in a specific area based on the given outlines created in the label map. With a set of special functions working, the constructure of interesting areas was automatically recognized. The painting should not include a single voxel outside of the region of interest, as the seeding is sensitive and will not respond well to outliers. Next, a gray sphere was extended by iteratively attempting to recognize and mark the partial pixels, which were the same as the pixels of the original label map in the intracranial cavity. Then, the entire endocranial boundary was reconstructed, and the algorithm was stopped. Due to the existence of the skull base foramen, foramen ovale, and foramen magnum, extra images might occur after the calculation. The Scissors tool can be used to remove extra images in the 3D window. After processing, the gray object represents a volumetric representation of the intracranial cavity, which includes brain tissue and non-brain tissue. Because of the distinct signal intensity thresholds of different tissues in the cranial cavity, the semiautomatic pixel threshold method was used to erase all non-brain tissues. The analysts interactively identified the threshold based on the density scale settings of the CT. Ultimately, brain volume was obtained (Figure 3). The CIBV was determined by subtracting the minimum brain volume (BV_max_) from the maximum brain volume (BV_min_) in the early course of aSAH. As a consequence, the CRBV was determined by CIBV/BV_min_ × 100% (Figure 4).

### Statistical Analysis

In our study, we divided all subjects into two groups based on the occurrence or absence of brain swelling after aSAH. According to the normality of the continuous variables, the unpaired Student's t-test or Mann–Whitney U-test was used for comparisons between the two groups. The Fisher exact test or χ^2^ test was used to analyze the categorical variables. Correlations were assessed using Spearman or Pearson rank correlation analyses. Some valuable variables were selected by univariate analysis (*p* < 0.05) and then incorporated into multivariate logistic regression analyses to identify the independent risk factors for unfavorable patient outcomes at 3 months after bleeding. Backward selection was performed using a multivariate logistic regression model to determine the independent predictors of poor prognosis, and the statistical results of the multivariate logistic regression analysis are reported as the odds ratio (OR) and 95% confidence interval (CI), and *p*-value. In the stratified analysis, the trisection interval was used to investigate the relationship between the degree of brain swelling and clinical outcomes (21). Receiver operating characteristic (ROC) curves were used to estimate the WFNS grade, HH grade, mFisher scale, and the score of CRBV (SCRBV) to predict unfavorable prognoses. All data were analyzed using SPSS version 25.0 statistical software, and the significance level was set at *p* < 0.05.

## Result

### Baseline Characteristics

In this study, the data for 327 patients were recorded, but only 130 patients met the inclusion criteria and were included in the analysis. Thirty-seven patients were over 60 years old. Ninety-five patients (73.08%) were female, and 35 patients (26.92%) were male. Seventy patients (53.84%) with aneurysms were clipped, 51 patients (39.23%) underwent embolization with coils, and nine patients (6.93%) did not receive surgical treatment. Seventy-one of the 130 patients (54.62%) underwent early surgical intervention (<72 h after bleeding). The mean BV_max_ was larger than the BV_min_ [1,181.10 ml (±107.17) vs. 1,120.02 ml (±113.41), *p* < 0.05]. The mean CIBV and CRBV were 62.28 ml (±59.99) and 5.81% (±5.98), respectively (Table 1).

**Table 1:** Study patient characteristics.

	***n* = 130**
**Baseline data** ***N*** **(%)**	
Age (>60 y)	37 (28.46)
Female	95 (73.08)
Smoking	28 (21.54)
Alcohol	27 (20.77)
Hypertension	55 (42.31)
Hyperlipidemia	24 (18.46)
Diabetes	8 (6.15)
**Clinical data** ***N*** **(%)**	
LOC	24 (18.46)
WFNS	
1	30 (23.08)
2	67 (51.54)
3	19 (14.62)
4	12 (9.23)
5	2 (1.54)
**HH**	
I	26 (20.00)
II	75 (57.69)
III	21 (16.15)
IV	4 (3.08)
V	4 (3.08)
**Radiological data** ***N*** **(%)**	
mFS	
1	35 (26.92)
2	46 (35.38)
3	40 (30.77)
4	9 (6.92)
**Aneurysm** ***N*** **(%)**	
Size of aneurysm (>10 mm)	8 (6.15)
Set of aneurysm (ACA)	82 (63.08)
Multiple aneurysms	28 (21.54)
**Treatment** ***N*** **(%)**	
Early surgery (Yes)	71 (54.62)
Clipping	70 (53.84)
Coiling	51 (39.23)
Others	9 (6.93)
**Complications** ***N*** **(%)**	
DCI	36 (27.69)
Hydrocephalus	20 (15.38)
**Volume in mL, mean (SD)**	
BV_max_	1181.10 ± 107.17
BV_min_	1120.02 ± 113.41
CIBV	62.28 ± 59.99
CRBV	5.81 ± 5.98
Blood in ambient cistern	5.40 ± 3.85
Blood in lateral ventricle	1.48 ± 2.15
Lateral ventricular volume	41.77 ± 22.87
**Outcomes, median, (IQR)**	
Hospital LOS^a^	19 (15,25)
mRS at 3 m^a^	2 (2,3)

*LOS, length of stay; LOC, loss of consciousness at ictus; IQR, interquartile range; mRS, modified Rankin Score; BV_max_, the maximum brain volume in the early course of aSAH; BV_min_, the minimum brain volume in the early course of aSAH; CIBV = BV_max_ - BV_min_; CRBV, Change Rate of Brain Volume; CRBV = (CIBV/BVmin) × 100%; DCI, Delayed Cerebral Ischemia; HH, Hunt-Hess scale; mFS, modified Fisher Scale; WFNS, World Federation of Neurosurgical Societies*.

a *Mann-Whitney U test*.

### Change Rate of Brain Volume and Primary Brain Swelling

Twenty-five of the 130 (19.23%) subjects were in the swelling group, and 105 (80.77%) were in the non-swelling group. Sixteen (64.00%) patients in the swelling group had a long history of hypertension, whereas 39 (37.14%) patients in the non-swelling group had hypertensive disease (*p* < 0.05). There was a significant difference between the groups in the WFNS (*p* < 0.05). The mean CRBV in the two groups, the swelling group, and non-swelling group, was normally distributed, with average values (±SD) of 11.87% (±6.84) and 4.37% (±4.77), respectively; there was a significant difference between the two groups (*p* < 0.01). The median mRS at 3 months after bleeding was 2 (2, 5) and 2 (2, 3) in the two groups (*p* < 0.01). Additional baseline characteristic details are shown in Table 2.

**Table 2:** Univariate analysis of characteristics of SAH patients.

**Variables**	**Non-brain swelling**	**Brain swelling**	***P*-value**
	**(*N* = 105)**	**(*N* = 25)**	
**Baseline data**			
Age (>60 y)	27 (25.71)	10 (40.00)	0.155
Female	75 (71.43)	20 (80.00)	0.385
Smoking	24 (22.86)	4 (16.00)	0.454
Alcohol	23 (21.90)	4 (16.00)	0.513
Hypertension	39 (37.14)	16 (64.00)	**0.015**
Hyperlipidemia	18 (17.14)	6 (24.00)	0.440
Diabetes	6 (5.71)	2 (8.00)	0.669
**Clinical data** ***N*** **(%)**			
LOC	17 (16.19)	7 (28.00)	0.179
WFNS			**<0.01**
1	28 (26.67)	2 (8.00)	
2	59 (56.19)	8 (32.00)	
3	12 (11.43)	7 (28.00)	
4	5 (4.76)	7 (28.00)	
5	1 (0.95)	1 (4.00)	
**HH**			0.103
I	25 (23.81)	1 (4.00)	
II	60 (57.14)	15 (60.00)	
III	15 (14.29)	6 (24.00)	
IV	3 (2.86)	1 (4.00)	
V	2 (1.90)	2 (8.00)	
**Radiological data** ***N*** **(%)**			
mFS			0.424
1	31 (29.52)	4 (16.00)	
2	36 (34.29)	10 (40.00)	
3	30 (28.57)	10 (40.00)	
4	8 (7.62)	1 (4.00)	
**Aneurysm** ***N*** **(%)**			
Size of aneurysm (>10 mm)	6 (5.71)	2 (8.00)	0.669
Set of aneurysm (ACA)	63 (60.00)	19 (76.00)	0.262
Multiple aneurysms	21 (20.00)	7 (28.00)	0.382
Early surgery (Yes)	55 (52.38)	16 (64.00)	0.294
**Treatment** ***N*** **(%)**			0.128
Clipping	57 (54.29)	13 (52.00)	
Coiling	43 (40.95)	8 (32.00)	
Others	5 (4.76)	4 (16.00)	
**Complications** ***N*** **(%)**			
DCI	28 (26.67)	8 (32.00)	0.592
Hydrocephalus	15 (14.29)	5 (20.00)	0.477
**Volume in mL, mean (SD)**			
BV_max_	1177.79 ± 110.71	1195.00 ± 91.54	0.473
BV_min_	1130.84 ± 114.27	1074.57 ± 99.44	**0.025**
CIBV	47.41 ± 45.90	124.76 ± 71.99	**<0.01**
CRBV	4.37 ± 4.77	11.87 ± 6.84	**<0.01**
Blood in ambient cistern	5.16 ± 3.65	6.40 ± 4.54	0.149
Blood in lateral ventricle	1.30 ± 1.97	2.24 ± 2.74	0.118
Lateral ventricular volume	41.63 ± 23.35	42.40 ± 21.21	0.880
**Outcomes, median (IQR)**			
Hospital LOS^a^	18 (14.23)	29 (19.43)	**<0.01**
mRS at 3 m^a^	2 (2.3)	2 (2.5)	**<0.01**

*Bold value indicates p < 0.05; LOS, length of stay; LOC, loss of consciousness at ictus; IQR, interquartile range; mRS, modified Rankin Score; BV_max_, the maximum brain volume in the early course of aSAH; BV_min_, the minimum brain volume in the early course of aSAH; CIBV = BV_max_ – BV_min_; CRBV, Change Rate of Brain Volume; CRBV = (CIBV/BVmin) × 100%; DCI, Delayed Cerebral Ischemia; HH, Hunt-Hess scale; mFS, modified Fisher Scale; WFNS, World Federation of Neurosurgical Societies*.

a *Mann-Whitney U test*.

### Change Rate of Brain Volume and Classical Grading

The CRBV was positively correlated with the length of stay in the hospital (Spearman ρ = 0.334; *p* < 0.001), blood in the ambient cistern (Pearson ρ = 0.269, *p* = 0.002), blood in the lateral ventricle (Pearson ρ = 0.278, *p* = 0.001), and lateral ventricular volume (Pearson ρ = 0.233, *p* = 0.008). The analysis of variance showed that there were significant differences among the varying degrees of the mFisher scale in CIBV, CRBV, blood in the ambient cistern, blood in the lateral ventricle, and lateral ventricular volume (Table 3). Similar results were found in the statistical analysis of HH grade (Supplementary Table 1) and WFNS grade (Supplementary Table 2). The higher the grade of the admission scale, the larger the CRBV (*p* < 0.01).

**Table 3:** Analysis of variance results of varying degree of mFisher score.

**Volume in mL, mean (SD)**	**Modified fisher scale**				***F***	***p***
	**1 (*n* = 35)**	**2 (*n* = 46)**	**3 (*n* = 40)**	**4 (*n* = 9)**		
Blood in ambient cistern	3.51 ± 2.21	5.04 ± 3.69	6.53 ± 4.31	9.56 ± 2.92	8.97	**<0.001**
Blood in lateral ventricle	0.63 ± 1.44	1.76 ± 2.18	1.50 ± 2.22	3.33 ± 2.83	4.65	**0.004**
Lateral ventricular volume	39.11 ± 21.68	37.80 ± 14.71	43.23 ± 28.06	66.00 ± 24.34	4.35	**0.006**
CIBV	38.00 ± 28.02	57.28 ± 58.44	66.67 ± 55.50	162.74 ± 79.46	13.56	**<0.001**
CRBV	3.36 ± 2.84	5.47 ± 5.82	6.04 ± 5.30	16.11 ± 8.37	14.24	**<0.001**

*Bold value indicates p <0.05, BV_max_, the maximum brain volume in the early course of aSAH; BV_min_, the minimum brain volume in the early course of aSAH; CIBV = BV_max_ – BV_min_, CRBV = (CIBV/BVmin) × 100%*.

### Change Rate of Brain Volume and Unfavorable Outcomes

Forty-two (32.31%) individuals in the study population had unfavorable outcomes with high-grade mRS at 3 months. Multivariate logistic regression analyses indicated that high-grade HH scale, high-grade mFisher scale, DCI, and CRBV were independent predictors of poor clinical outcomes. The model showed that for every 1% increase in the CRBV, the odds of a poor outcome increased by a factor of 1.236 (95% CI = 1.056–1.446) after adjusting for age, hypertension, LOC, WFNS, HH, mFisher, blood in the ambient cistern, DCI, hydrocephalus, and lateral ventricular volume; additional details are shown in Table 4. Stratified analysis showed that the odds of poor clinical outcomes increased with increasing CRBV. A larger CRBV was associated with poor clinical outcomes compared with the values in the lowest tertile (Table 5).

**Table 4:** Predictors of outcome.

**Total (*N* = 130)**	**Good outcome (*N* = 88)**	**Severe disability (*N* = 42)**	**Odds ratio (95% CI)**	***P* Univariable**	**Odds ratio (95% CI)**	***P* Multivariable^a^**
**Baseline data** ***N*** **(%)**						
Age (>60 y)	20 (22.73)	17 (40.48)	2.312 (1.047 ~ 5.107)	**0.038**	2.167 (0.591 ~ 7.950)	0.243
Female	61 (69.32)	34 (80.95)	1.881 (0.770 ~ 4.597)	0.166		
Smoking	15 (17.05)	13 (30.95)	2.182 (0.925 ~ 5.147)	0.075		
Alcohol	16 (18.18)	11 (26.19)	1.597 (0.665 ~ 3.833)	0.295		
Hypertension	31 (35.23)	24 (57.14)	2.452 (1.156 ~ 5.198)	**0.019**	2.573 (0.773 ~ 8.560)	0.123
Hyperlipidemia	16 (18.39)	8 (19.05)	1.044 (0.407 ~ 2.678)	0.928		
Diabetes	6 (6.82)	2 (4.76)	0.683 (0.132 ~ 3.538)	0.650		
**Clinical data** ***N*** **(%)**						
LOC	12 (13.63)	12 (28.57)	2.533 (1.025 ~ 6.262)	**0.044**	1.646 (0.344 ~ 7.878)	0.533
WFNS			1.832 (1.213 ~ 2.769)	**0.004**	0.576 (0.267 ~ 1.242)	0.159
1	24 (27.27)	6 (14.29)				
2	48 (54.55)	19 (45.24)				
3	10 (11.36)	9 (21.43)				
4	6 (6.82)	6 (14.29)				
5	0 (0.00)	2 (4.76)				
HH			5.287 (2.584~10.818)	**<0.001**	3.096 (1.159~8.268)	**0.024**
I	24 (27.27)	2 (4.76)				
II	58 (65.91)	17 (40.48)				
III	5 (5.68)	16 (38.10)				
IV	0 (0.00)	4 (9.52)				
V	1 (1.14)	3 (7.14)				
**Radiological data** ***N*** **(%)**						
mFS			3.667 (2.144 ~ 6.273)	**<0.001**	3.120 (1.345 ~ 7.235)	**0.008**
1	32 (36.36)	3 (7.14)				
2	35 (39.77)	11 (26.19)				
3	20 (22.73)	20 (47.62)				
4	1 (1.14)	8 (19.05)				
**Aneurysm** ***N*** **(%)**						
Size of aneurysm (>10 mm)	3 (3.41)	5 (11.90)	3.829 (0.869 ~ 16.862)	0.076		
Set of aneurysm (ACA)	53 (60.23)	29 (69.05)	0.779 (0.426 ~ 1.426)	0.419		
Multiple aneurysms	19 (21.59)	9 (21.43)	0.990 (0.405 ~ 2.424)	0.983		
Early surgery (Yes)	50 (56.82)	21 (50.00)	0.760 (0.364 ~ 1.589)	0.466		
**Treatment** ***N*** **(%)**			1.157 (0.743 ~ 1.802)	0.518		
Clipping	29 (32.95)	22 (52.38)				
Coiling	29 (32.95)	22 (52.38)				
Others	7 (7.95)	2 (4.76)				
**Complications** ***N*** **(%)**						
DCI	14 (15.91)	22 (52.38)	5.814 (2.529 ~ 13.367)	**<0.001**	10.617 (2.927 ~ 38.503)	**<0.001**
Hydrocephalus	9 (10.23)	11 (26.19)	3.115 (1.176 ~ 8.249)	**0.022**	2.966 (0.543 ~ 16.212)	0.210
**Volume in mL, mean (SD)**						
BV_max_	1172.94 ± 107.01	1198.21 ± 106.76	1.002 (0.999 ~ 1.006)	0.21		
BV_min_	1132.24 ± 107.27	1094.43 ± 122.71	0.997 (0.994 ~ 1.000)	0.078		
CIBV	40.56 ± 27.82	107.79 ± 80.93	1.026 (1015 ~ 1.038)	**<0.001**	1.026 (1.007 ~ 1.045)	**0.007**
CRBV	3.65 ± 2.64	10.36 ± 8.18	1.293 (1.153 ~ 1.449)	**<0.001**	1.236 (1.056 ~ 1.446)	**0.008**
Blood in ambient cistern	4.76 ± 3.35	6.74 ± 4.49	1.142 (1.035 ~ 1.259)	**0.008**	0.882 (0.726 ~ 1.071)	0.204
Blood in lateral ventricle	1.41 ± 2.07	1.64 ± 2.35	1.051 (0.889 ~ 1.242)	0.563		
Lateral ventricular volume	38.01 ± 20.08	49.67 ± 26.39	1.023 (1.006 ~ 1.040)	**0.009**	1.017 (0.988 ~ 1.047)	0.259

*Bold value indicates p < 0.05; LOS, length of stay; LOC, loss of consciousness at ictus; IQR, interquartile range; mRS, modified Rankin Score; BV_max_, the maximum brain volume in the early course of aSAH; BV_min_, the minimum brain volume in the early course of aSAH; CIBV = BV_max_ – BV_min_; CRBV = (CIBV/BVmin) × 100%; DCI, Delayed Cerebral Ischemia; HH, Hunt-Hess scale; mFS, modified Fisher Scale; WFNS, World Federation of Neurosurgical Societies*.

a *Mann-Whitney U test. ^a^Adjusted for age, LOC, hypertension, WFNS, HH, mFisher, DCI, Hydrocephalus, CRBV, Blood in ambient cistern, Lateral ventricular volume*.

**Table 5:** Outcome association with stratified analysis of the CRBV.

**Tertiles**	**CRBV (%)**	**Good outcome**	**Severe disability**	**Crude OR**	**Adjusted OR**
		**(*N* = 88)**	**(*N* = 42)**	**(95% CI)**	**(95% CI)^a^**
Tertile 1	CRBV ≤ 2.6	41	2	1	1
Tertile 2	2.6 < CRBV ≤ 5.3	29	15	10.603 (2.250 ~ 49.966)	8.578 (1.488 ~ 49.441)
Tertile 3	CRBV ≥ 5.3	18	25	28.472 (6.084 ~ 133.238)	17.454 (2.866 ~ 106.302)
*P* for trend				**<0.001**	**0.008**

*CRBV = (CIBV/BV_min_) × 100%*,

a *adjusted for age, hypertension, LOC, WFNS grade, HH grade, mFisher scale, DCI, Hydrocephalus, Blood in ambient cistern, Lateral ventricular volume. Bold value indicates p < 0.05*.

### Prognostic Value of Score of Change Rate of Brain Volume for Unfavorable Outcomes

**Figure 5:**
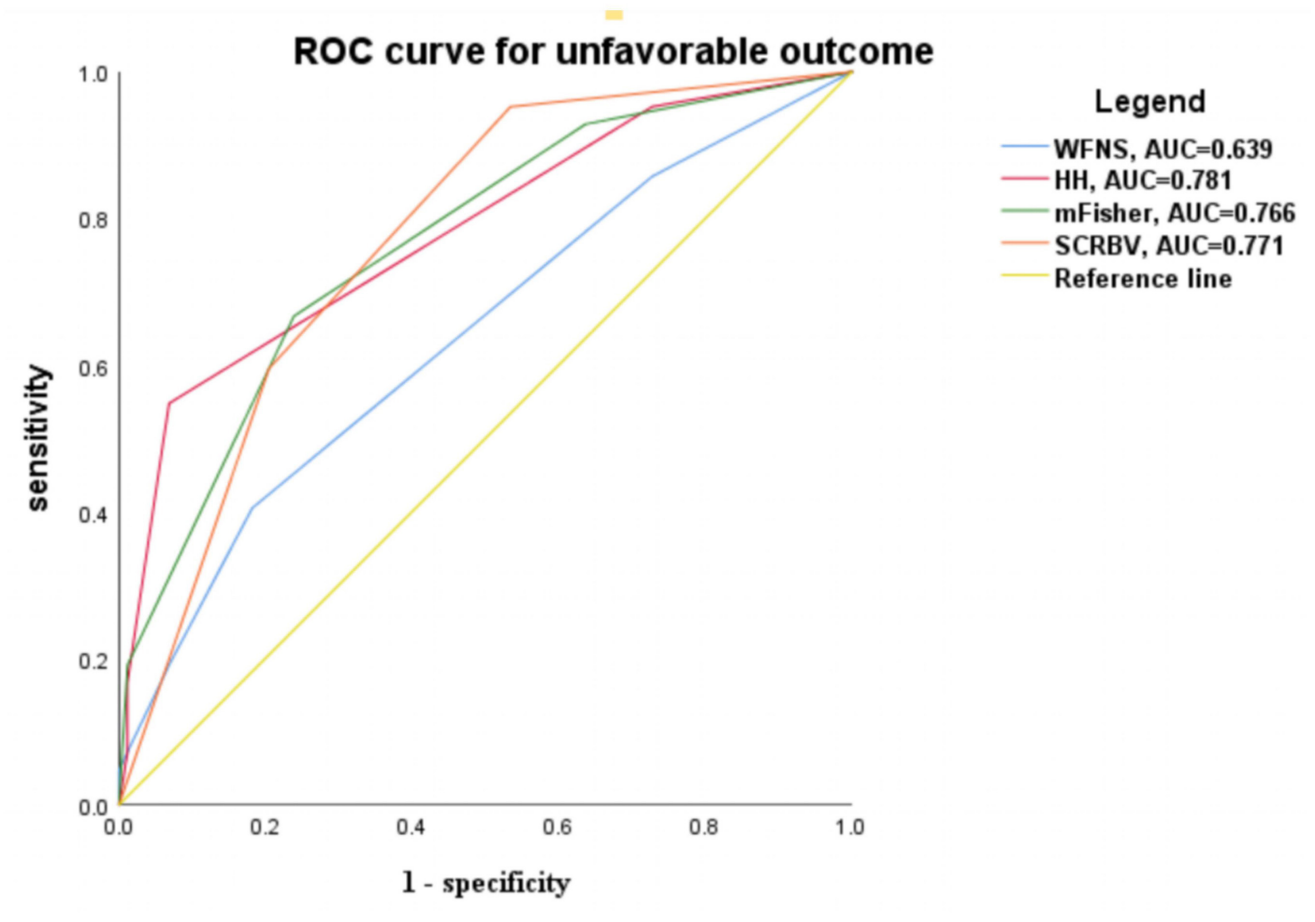
ROC curve for predicting the unfavorable outcome according to the clinical and radiographic grades. *WFNS*, World federation for neurological surgeons grade; *HH*, Hunt-Hess grade; *mFisher*, modified Fisher score; *SCRBV*, Score of Change Rate of Brain Volume.

We developed a scoring system, namely, the score of CRBV (SCRBV), based on hierarchical analysis: a score of 1 was assigned if the CRBV of the patient was in the lower tertile, 2 if the CRBV was in the middle tertile, and 3 if the CRBV was in the upper tertile; the minimum and maximum scores of the system were 1 and 3, respectively. In our study, 43 subjects (33.07%) received 1 point, 44 subjects (33.86%) received 2 points, and 43 subjects (33.07%) received 3 points. ROC curve analysis showed that admission HH grade (AUG = 0.781, 95% CI = 0.693–0.870), admission mFisher scale (AUG = 0.766, 95% CI = 0.679–0.853), admission WFNS score (AUG = 0.639, 95% CI = 0.536–0.743), and SCRBV (AUG = 0.771, 95% CI = 0.689–0.852) had diagnostic performance for predicting unfavorable outcomes (Figure 5).

## Discussion

In our study, we mainly found that the CRBV is associated with the poor outcomes of patients with aSAH, and we provided non-invasive, semiautomated, inexpensive measurements using 3D-Slicer to quantify the degree of brain swelling after aSAH. Two interesting results are recognized: (1) the CRBV in the swelling group was larger than that in the non-swelling group, and (2) subjects with a smaller CRBV had a better clinical outcome at 3 months follow-up. Such results suggest that early primary brain swelling after aSAH is a crucial target for timely treatment, and the CRBV may be an alternative marker for therapeutic efficacy in aSAH treatment.

The qualitative criteria of primary brain swelling are helpful for the management and treatment of patients, but at the same time, they have certain limitations. There are varying degrees of severity of primary brain swelling, and it does not have a binary outcome; therefore, the dichotomization of brain swelling hinders its further research. Quantitative methods have been used to study diseases in many fields (22, 23), and combining both quantitative and qualitative criteria could help clinicians in the disease-treatment process (24). Brain swelling is a complex pathophysiological process, and dysfunction of cerebral blood flow autoregulation may be the initiating factor for the occurrence and development of brain swelling (25). In this study, the CRBV in the brain swelling group was larger than that in the non-swelling group. This could mean that the dysfunction of cerebral blood flow autoregulation that occurred in the swelling group was much more serious than that in the non-swelling group. The brain can experience reactive hyperemia after aSAH, which can result in an increase in brain volume (26, 27). Therefore, early primary brain swelling may be an indication of hyperemia; hence, we suggest that further studies of brain swelling should include assessments of blood flow, such as CTP.

We found that the BV_max_ value was not significantly different between the swelling group and non-swelling group, but the CRBV was significantly different between the two groups (*p* < 0.05). This may be related to the physiological anatomy of the brain: the changes in total BV, gray matter volume, and white matter volume reach their maximum during young age, and then these volumes gradually decrease, and sulcal volumes increase (28). In addition, because of the limitation of the skull, the volume of the cranial cavity is constant. Therefore, it is reasonable that there was no significant difference in the BV_max_ between the two groups. So, if we merely consider the BV at a certain time point during the course of the SAH instead of taking into account the dynamic changes in BV, the results may be affected by the physiological anatomy of the brain and may overlook some underlying changes in the brain parenchyma. These potential harmful pathophysiological processes may be manifested by increased brain volume, which can worsen the condition of the patients and force them to undergo DHC. Although DHC improves the survival rate of patients, the overall quality of life after DHC is very poor, and the long-term quality of life of DHC is unknown (29).

Our results are consistent with our prediction that there are significant differences among varying degrees of the mFisher scale in terms of blood in the ambient cistern and blood in the lateral ventricle. The higher the grade of the admission mFisher scale, the larger the values of these variables. It is reasonable that this finding can be explained by the qualitative diagnosis of the mFisher scale (30). Although the mFisher scale is a reliable and widely accepted radiologic grading system for predicting delayed cerebral ischemia (DCI), this scale focuses on predicting obvious vascular vasospasms based on the amount of blood in the basal cisterns and the intraventricular system. Our findings revealed that the amount of bleeding was related to the CRBV. This may be attributed to the disruption of brain tissue in the early stage after SAH, which may be at the level of microcirculation (31). The CRBV may reflect additional changes in brain physiology that represent microcirculation changes.

The model showed that a larger CRBV was related to worse outcomes after adjusting for age, hypertension, LOC, WFNS, HH, mFisher, blood in the ambient cistern, DCI, hydrocephalus, and lateral ventricular volume, and emphasized that the CRBV may be an independent risk factor for poor clinical outcomes. Moreover, stratified analysis showed that the odds of worse clinical outcomes increased with increasing CRBV. A larger CRBV was associated with poor clinical outcomes compared with the values in the lower tertile. Under normal physiological conditions, brain tissue has the function of compliance and automatic regulation, which can maintain the balance and stability of cerebral perfusion pressure (CPP) and intracranial pressure (ICP) by contracting or relaxing blood vessels and adjusting cerebral blood flow. Under the pathological conditions, the change in brain tissue compliance and the decompensation of brain volume increase may lead to an increase in intracranial pressure and a decrease in perfusion pressure, thereafter, deteriorating the neurological dysfunction of patients (32). The CRBV may be related to brain tissue compliance. The larger the CRBV, the greater the probability of the decompensation of brain volume. Brain CT scans of all subjects were evaluated, and the CRBV was a kind of relative value of brain volume changes in the early course of aSAH. The advantage of assessing the CBRV is that brain swelling can be observed dynamically, and it may be helpful for the early diagnosis and timely treatment of patients after aSAH.

After some studies encountered difficulties in the treatment and prevention of vasospasm, early brain injury (EBI) has attracted more attention (33, 34). EBI reflects the initial clinical presentation and is associated with secondary complications and poor clinical prognosis (35). Many important pathological mechanisms have been recognized within minutes of bleeding, including dysfunction of cerebral blood flow perfusion, cerebral ischemia, and abnormal brain metabolism, which can cause an increase in brain volume (27, 36). It has been confirmed in animal experiments that the brain volume increases in the early stage after hemorrhage (37). Recently, brain swelling, which is sometimes recognized as global cerebral edema (GCE), has been proven to be an available radiographic marker of EBI (38). The diagnosis of GCE was based on a dichotomized qualitative assessment (39), and it may reflect the fact that this qualitative radiographic criteria tend to identify severe swelling cases. In fact, GCE is not an all or none phenomena but rather a process with degrees of severity. Recently, Yuan et al. quantified GCE after aSAH by inventing the automated assessment of selective sulcal volume (SSV) (40). SSV was defined as total ml of sulcal volumes on axial CT slices above the most cranial section of the lateral ventricles, instead of the whole brain volume change. On the contrast, we used quantitative analysis method to calculate CRBV, which can better represent the severity of the swelling. HH and WFNS are the most widely used clinical grading scales for predicting prognosis by evaluating the signs and symptoms of patients. However, these scoring systems cannot take into account the amount of blood. With the development of CT, some radiographic scales have been established. The mFisher scale was established by quantifying the amount of bleeding. However, none of these scales focus on EBI. In our study, the ROC curve showed that admission HH grade, admission mFisher scale, WFNS, and SCRBV had diagnostic performance for predicting unfavorable outcomes accordingly in the clinic. It is suggested that when the increase in brain volume exceeds a certain threshold, the risk of poor prognosis is significantly increased. These results indicate that CRBV can better reflect the clinical severity of patients with aSAH and can be used as an imaging marker for the diagnosis of EBI after aSAH.

In the clinic practice, secondary brain swelling caused by hematoma, infarction, and hydrocephalus can be simply distinguished by CT. However, it is worth noting that pseudohypoxic brain swelling, a rare but potentially life-threatening complication, may be confused with primary brain swelling after SAH (41). Its occurrence and development are related to excessive drainage of cerebrospinal fluid (42). The timely cessation of any operation that causes a reduction in cerebrospinal fluid and placing the patient in the Trendelenburg position can relieve the symptoms of the patients. From the aspect of neuroimaging, low density (CT) and high signal (MRI) may be present in the basal ganglia, thalamus, cerebellum, and other anatomical areas (43). Therefore, better identification will allow the further exploration of primary brain swelling.

Although we controlled for conceivable influences, some weaknesses should be considered in our study. First, our study is bound by the limitations of a single observational study; however, the imaging operators were blinded to the patient's clinical information during the imaging measurements to eliminate bias. Second, compared with CT, MRI is a more accurate imaging tool for the assessment of brain volume. However, MRI is inconvenient for severe aSAH patients. Instead, almost every aSAH patient underwent multiple CT scans during hospitalization, and the CRBV is a direct measurement of the degree of brain swelling.

## Conclusion

In summary, there was a remarkable difference in the CRBV between the swelling group and non-swelling group. The CRBV is quantified by 3D-Slicer, which can be used as a volumetric representation of the degree of primary brain swelling. The CRBV may serve as a surrogate biomarker of EBI after bleeding, which can predict the clinical prognoses of the patients. A larger SCRBV in the early stage of aSAH was related to worse prognosis compared with the lower SCRBV.

## Data Availability Statement

The raw data supporting the conclusions of this article will be made available by the authors, without undue reservation.

## Ethics Statement

The studies involving human participants were reviewed and approved by the Institutional Review Board of The First Affiliated Hospital of Chongqing Medical University. The Ethics Committee waived the requirement of written informed consent for participation.

## Author Contributions

BQ handled the conceptualization, methodology, validation, software, formal analysis, data curation, quantitative analysis, and writing of the original draft. YX and JZ were in charge of the investigation and quantitative analysis. RX, ZG, CC, LJ, and YW contributed to the acquisition of resources, the review, and writing of the manuscript. XS and ZH did the conceptualization, methodology, acquisition of resources, data curation, funding acquisition, review, editing, and writing of the manuscript. All authors contributed to the article and approved the submitted version.

## Funding

The National Natural Science Foundation of China supports this research (Grant No. 82071332). All authors contributed to the article and approved the submitted version.

## Conflict of Interest

The authors declare that the research was conducted in the absence of any commercial or financial relationships that could be construed as a potential conflict of interest.

## Publisher's Note

All claims expressed in this article are solely those of the authors and do not necessarily represent those of their affiliated organizations, or those of the publisher, the editors and the reviewers. Any product that may be evaluated in this article, or claim that may be made by its manufacturer, is not guaranteed or endorsed by the publisher.
