# Supplementary material for: Increase in Brain Volume After Aneurysmal Subarachnoid Hemorrhage Leads to Unfavorable Outcome: A Retrospective Study Quantified by CT Scan
Source: Front Neurol. 2021 Oct 8;12:654419. doi: 10.3389/fneur.2021.654419 (PMC8531099; doi:10.3389/fneur.2021.654419)
Supplement: Supplementary file 1 [file Data_Sheet_1.doc]

|  | [**Supplemental Table 1 Analysis of variance results of varying degree of Hunt-Hess**](#结果表2) **grade** | | | | | | | |
| --- | --- | --- | --- | --- | --- | --- | --- | --- |
| Volume in mL, mean (SD) | | Hunt-Hess grade | | | | | F | p |
|  |  | 1 (n=26) | 2 (n=75) | 3 (n=21) | 4 (n=4) | 5 (n=4) |  |  |
| Blood in ambient cistern | | 4.46±3.31 | 5.27±3.89 | 6.33±3.94 | 6.25±3.20 | 8.25±5.74 | 1.328 | 0.263 |
| Blood in lateral ventricle | | 1.58±2.48 | 1.27±1.93 | 1.43±2.13 | 2.25±2.63 | 4.50±2.38 | 2.384 | 0.055 |
| Lateral ventricular volume | | 36.00±14.72 | 40.91±21.50 | 46.10±31.79 | 64.25±32.15 | 50.50±17.60 | 1.781 | 0.137 |
| CIBV | | 41.42±30.75 | 48.30±41.64 | 84.33±52.94 | 182.65±110.82 | 223.90±83.49 | 23.355 | **<0.001** |
| CRBV | | 3.66±2.72 | 4.50±4.28 | 7.97±5.71 | 18.64±11.47 | 20.41±7.79 | 21.007 | **<0.001** |
| **Bold value** indicates p<0.05, ***BV_max_*** the maximum brain volume in the early course of aSAH, ***BV_min_*** the minimum brain volume in the early course of aSAH, ***CIBV*** = ***BV_max_*** - ***BV_min_***, CRBV=(CIBV/BVmin) × 100%. | | | | | | | | |

|  | [**Supplemental Table 2 Analysis of variance results of varying degree of WFNS**](#结果表2) **grade** | | | | | | | |
| --- | --- | --- | --- | --- | --- | --- | --- | --- |
| Volume in mL, mean (SD) | | WFNS grade | | | | | F | p |
|  |  | 1.0(n=30) | 2.0(n=67) | 3.0(n=19) | 4.0(n=12) | 5.0(n=2) |  |  |
| Blood in ambient cistern | | 5.13±4.08 | 5.15±3.76 | 5.53±4.17 | 6.33±2.93 | 11.00±2.83 | 1.362 | 0.251 |
| Blood in lateral ventricle | | 1.43±1.96 | 1.39±2.16 | 1.32±2.16 | 1.75±2.38 | 5.50±0.71 | 1.895 | 0.115 |
| Lateral ventricular volume | | 36.60±17.84 | 41.94±22.55 | 43.32±28.50 | 48.00±26.56 | 62.00±14.14 | 1.020 | 0.400 |
| CIBV | | 49.70±46.97 | 49.98±46.94 | 86.09±79.91 | 93.29±52.95 | 250.94±28.48 | 9.513 | **<0.001** |
| CRBV | | 4.82±5.43 | 4.60±4.78 | 7.99±7.42 | 8.70±5.33 | 23.59±2.15 | 8.087 | **<0.001** |
| **Bold value** indicates p<0.05, ***BV_max_*** the maximum brain volume in the early course of aSAH, ***BV_min_*** the minimum brain volume in the early course of aSAH, ***CIBV*** = ***BV_max_*** - ***BV_min_***, CRBV=(CIBV/BVmin) × 100%. | | | | | | | | |
