# Supplementary figures and images for: Increase in Brain Volume After Aneurysmal Subarachnoid Hemorrhage Leads to Unfavorable Outcome: A Retrospective Study Quantified by CT Scan
Source: Front Neurol. 2021 Oct 8;12:654419. doi: 10.3389/fneur.2021.654419 (PMC8531099; doi:10.3389/fneur.2021.654419)

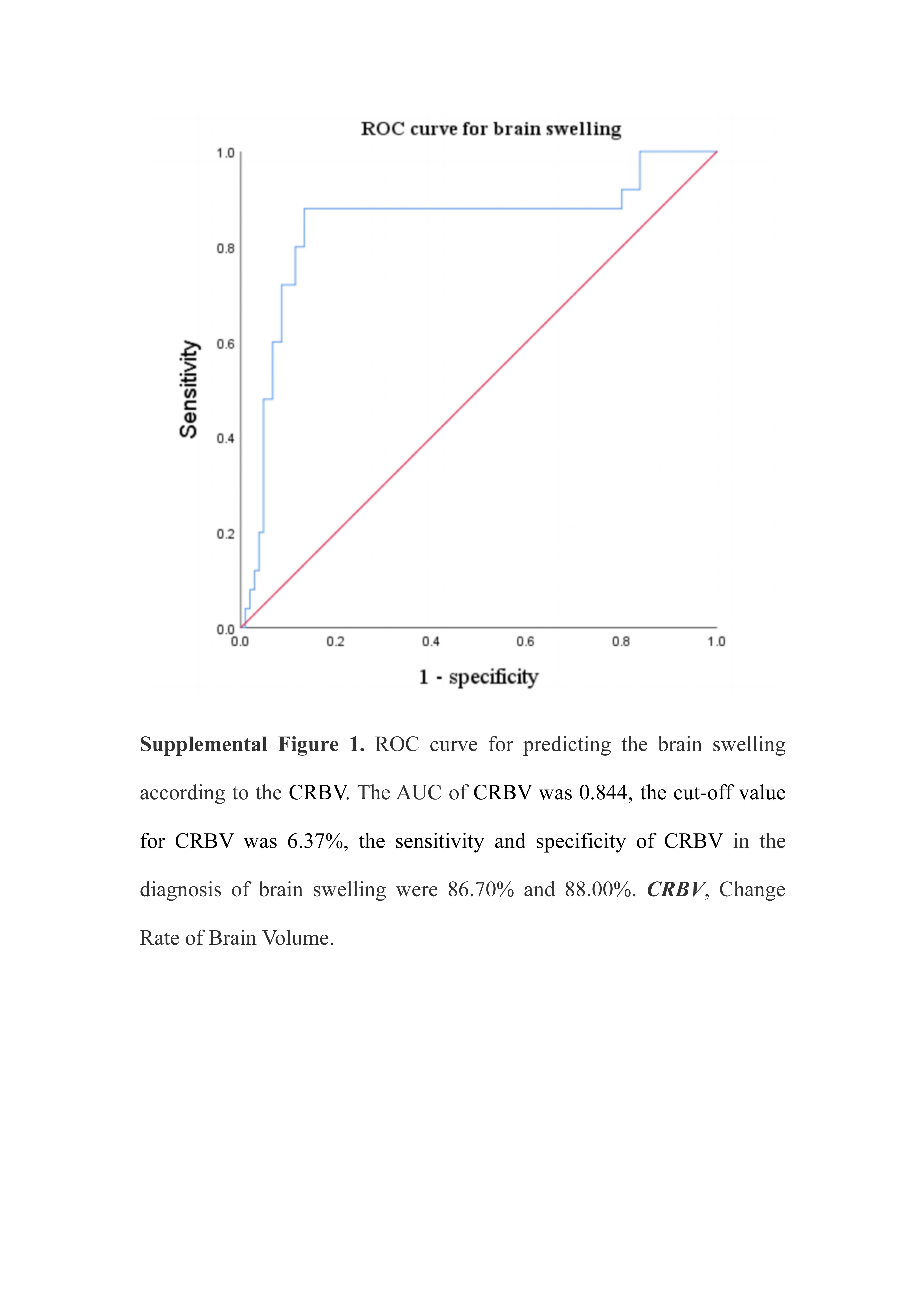

Supplement: Supplementary file 2 [file Image_1.JPEG]
